# Treatment Advances in Sepsis and Septic Shock: Modulating Pro- and Anti-Inflammatory Mechanisms

**DOI:** 10.3390/jcm12082892

**Published:** 2023-04-15

**Authors:** Adriana Marques, Carla Torre, Rui Pinto, Bruno Sepodes, João Rocha

**Affiliations:** 1 Research Institute for Medicines (iMed.ULisboa), Faculty of Pharmacy, Universidade de Lisboa, 1649-003 Lisbon, Portugal; adriana.marques1@campus.ul.pt (A.M.); carla.torre@ff.ulisboa.pt (C.T.); rapinto@ff.ulisboa.pt (R.P.); bsepodes@ff.ulisboa.pt (B.S.); 2 Joaquim Chaves Saúde, Joaquim Chaves Laboratório de Análises Clínicas, Miraflores, 1495-069 Algés, Portugal

**Keywords:** sepsis, septic shock, inflammation, immunomodulation, personalized medicine

## Abstract

Sepsis is currently defined as a life-threatening organ dysfunction caused by a dysregulated host response to infection, and it affects over 25 million people every year. Even more severe, septic shock is a subset of sepsis defined by persistent hypotension, and hospital mortality rates are higher than 40%. Although early sepsis mortality has greatly improved in the past few years, sepsis patients who survive the hyperinflammation and subsequent organ damage often die from long-term complications, such as secondary infection, and despite decades of clinical trials targeting this stage of the disease, currently, no sepsis-specific therapies exist. As new pathophysiological mechanisms have been uncovered, immunostimulatory therapy has emerged as a promising path forward. Highly investigated treatment strategies include cytokines and growth factors, immune checkpoint inhibitors, and even cellular therapies. There is much to be learned from related illnesses, and immunotherapy trials in oncology, as well as the recent COVID-19 pandemic, have greatly informed sepsis research. Although the journey ahead is a long one, the stratification of patients according to their immune status and the employment of combination therapies represent a hopeful way forward.

## 1. Introduction

Since 2016, sepsis has been defined as “life-threatening organ dysfunction caused by a dysregulated host response to infection” and is represented as an increase of 2 or more points in the Sequential Organ Failure Assessment (SOFA) score [1]. In these patients, a dysregulated immune response can lead to an exaggerated pro-inflammatory process, immunosuppression, and/or persistent immune disruption [2]. Even more severe, septic shock is currently defined as a “subset of sepsis in which underlying circulatory and cellular metabolism abnormalities are profound enough to substantially increase mortality” and is associated with hospital mortality rates higher than 40% [1]. Patients with septic shock are characterized by persistent hypotension despite adequate volume resuscitation, the need for vasopressor therapy, and lactate >2 mmol/L [1,3]. The resulting metabolic dysfunction and inadequate tissue perfusion may ultimately lead to multiorgan failure and death [4,5].

Data from 2018 show sepsis affects approximately 27–30 million people worldwide, resulting in 6–9 million deaths every year [5], and while the real incidence and mortality attributed to sepsis are unknown, there is little doubt that it represents a significant challenge [1,3,6]. New treatment protocols and advancements in therapeutic approaches shifted the paradigm towards a more chronic, immunosuppressive stage of the disease [7], responsible for much of the later-stage morbidity and mortality. Importantly, epidemiological data on the incidence and mortality of sepsis is typically extrapolated from high-income countries, making it difficult to determine the true burden of this syndrome [6].

In the short term, sepsis survivorship is increasing [8,9]. The Surviving Sepsis Campaign (SSC), recently updated in 2021, provides evidence-based guidelines on identifying and treating these patients [10], which have contributed to reducing in-hospital mortality [3]. These guidelines provide guidance on the administration of antibiotics, appropriate source control interventions, fluid and vasopressor therapy, and other adjuvant measures. However, even though 50% of sepsis survivors recover once they are discharged from the hospital, one-third die within the next year, and one-sixth develop persistent cognitive impairment [11,12,13]. In the coming years, late-sepsis mortality is expected to increase [14] and disproportionately affect the growing elderly population, who often have weakened immune systems and other comorbidities [15]. No sepsis-specific therapies exist, and new approaches are urgently needed [16,17].

As decades of clinical trials targeting hyperinflammation have been somewhat unsuccessful and new pathophysiological mechanisms have been uncovered, the focus of more recent research has shifted to the immunosuppressive phase of sepsis and novel immunomodulatory therapies. Remarkably, anti-inflammatory therapies such as cytokine blockers have recently shown tremendous success in severe COVID-19 [18], a sepsis-like illness characterized by an imbalanced immune response [19]. Since the voluntary withdrawal of the marketing authorization of drotrecogin alpha (activated) (DAA) due to unsuccessful results of the post-authorization measures delineated for this product [20], no other therapies have been approved specifically for the indication of sepsis or septic shock [21].

Patients with sepsis typically present with a highly dysregulated immune system that fluctuates from a state of excessive inflammation to one of immunosuppression. In the case of sepsis, clinically relevant biomarkers must correctly identify each patient’s individual immune balance [22]—adequate stratification is needed to ensure the correct patient is receiving the appropriate treatment at the right time. Through transcriptomic profiling, two different immune phenotypes have been recognized in sepsis [23]: sepsis response signature (SRS)1 or SRS2. While the SRS2 phenotype is relatively immunocompetent, SRS1 identified patients with a more immunosuppressed profile, characterized by T-cell exhaustion, endotoxin tolerance, and low leukocyte HLA-DR expression. Similar results have also been described by Wang et al. [24]. However, of the 258 biomarkers that have been identified over the past decade [25,26], none have shown the necessary sensitivity and specificity to be used in routine clinical practice.

This scoping review aims to examine current research regarding the modulation of the host response to sepsis and septic shock and integrate the underlying pathophysiological mechanisms with different therapeutic strategies and potential biomarkers to better guide treatment. Given the broad and exploratory purpose of this review, we followed a scoping review methodology [27,28]: our search for treatment strategies and pathophysiological changes focused on articles published in the last 10 years and written in English. Selected articles were screened, and the reference lists of all the included studies were searched for any relevant articles we may have missed in the electronic searches. After mapping out the major causative mechanisms and corresponding treatment options, we researched ways to optimize sepsis management (e.g., biomarkers) and integrated those into the manuscript. The key findings of this review are summarized and critically examined in the Discussion portion of the text. With this scoping review, we have successfully assessed the extent of current evidence regarding immune modulation in sepsis and septic shock and highlighted research gaps in this topic. An overview of current clinical trials and future steps is also provided.

## 2. Modulating the Host Response to Sepsis

As multiple studies have shown that the immune response is not linear, revised models of sepsis pathophysiology have been proposed, with Persistent Inflammation, Immunosuppression, and Catabolism Syndrome (PICS) being the most relevant one [29,30]. In these patients, immunosuppression coexists with low-grade inflammation, making it difficult to target either phase of the immune response. Since traditional treatment strategies have been insufficient to curb long-term mortality, immunoadjuvant therapy has emerged as a promising way forward and research focus has largely shifted into targeting specific mechanisms of sepsis pathophysiology.

Following the methodology described in Section 1 of this review, the coming sections summarize the major alterations in the host response during sepsis and provide a rationale for potential therapeutic interventions. A compilation of recent clinical trials on the subject is provided in Table 1 and Table 2. We included completed (Table 1) and ongoing (Table 2) interventional studies indexed on ClinicalTrials.gov that both started in the last ten years and studied biological interventions not already discussed in the SSC guidelines (interventions such as antimicrobials or extracorporeal blood purification were excluded from this search). We focused our search on therapeutic strategies aiming at treating sepsis or septic shock and excluded those aiming at prevention. Clinical trials that were terminated or suspended, rather than completed, or with unknown or withdrawn status, were also excluded. Other clinical trial databases, such as Medline and the European and WHO registries, were also searched, in order to identify any missing trials that fit the pre-specified criteria. Trials identified in these databases were then searched in ClinicalTrials.gov and are included in Table 1 and Table 2.

### 2.1. The Complement System

After infection, the presence of pathogen-associated molecular patterns induces the expression of pro-inflammatory molecules and activation of the coagulation cascade and of the complement system [3,4]. C5a and its receptors, C5aR and C5aR2, have emerged as promising targets for sepsis therapy. Several authors have described the effect of C5a blockade in numerous animal models of inflammation, and this intervention has generally improved outcomes [61,62].

For example, in a murine model of sepsis induced by cecal ligation and puncture (CLP), anti-C5a treatment lowered thymocyte apoptosis by 80%. Furthermore, it also decreased serum levels of IL-6 and TNF-α [61], and reduced the incidence of multiorgan failure [62]. CaCP29, a humanized monoclonal antibody (mAb) developed by InflaRx, proved itself safe and well-tolerated in a dose escalation Phase I clinical trial in healthy human subjects (NCT01319903) [63,64]. A phase II clinical trial for this mAb (NCT02246595, Table 1), also referred to as IFX-1 or vilobelimab, has also been completed, but no results have been published [31,65].

C3a, on the other hand, induces platelet aggregation and leukocyte recruitment [66]. Compstatin is a cyclic peptide that inhibits C3 convertase-mediated cleavage of C3, thus limiting C3a and C3b formation. In a non-human primate model of *Escherichia coli* sepsis, compstatin administration reduced fibrinogen and platelet consumption, kidney injury, and improved systemic blood pressure [66]. However, C3b is key for bacteria opsonization and phagocytosis, and therapeutic strategies targeting C3 may hinder the normal in vivo response to infection [62].

### 2.2. Coagulation and Endothelial Activation

Both inflammatory cytokines and complement peptides profoundly activate the coagulation system, resulting in a shift towards a pro-coagulant state of the endothelium [3]. This leads to endothelial barrier disruption: tissue factor, thrombin, and other clotting factors activate protease-activated receptors (PARs) [65], which play a pivotal role in sepsis and can induce platelet aggregation, endothelial cell contraction, and vascular hyperpermeability [61,65]. Vorapaxar, a reversible competitive antagonist of PAR-1, reduced endothelial activation, pro-inflammatory cytokine release, and LPS-induced coagulation activation in a human endotoxemia model (NCT02875028, Table 1) [33,67], an in vivo model of systemic inflammation in which lipopolysaccharide is injected or infused intravenously in healthy volunteers.

### 2.3. Immunoparalysis

Immunosuppressed patients in the ICU typically show impaired immune cell function, which often culminates in decreased production of pro-inflammatory cytokines and other effector molecules, a condition commonly referred to as immunoparalysis or endotoxin tolerance [2,22]. For example, there is a marked decrease in the production of interferon-γ [68], which is vital for the host response against intracellular pathogens [14,68]. Administration of IFN-γ has been proposed as an adjunctive therapy in sepsis, as it substantially activates monocytes and enhances their antigen-presenting capacity [14,69]. In 1997, eight out of nine patients treated with IFN-γ recovered from sepsis, although two of them relapsed after IFN-γ discontinuation [70]. In this study, IFN-γ was able to restore monocyte production of TNF-α and HLA-DR expression in a dose-dependent manner. These results led to a new perspective on sepsis management, and research on immune stimulation skyrocketed [70]. Following up on a pilot study from 2012 (NCT01649921) aiming to investigate the effects of IFN-γ therapy in sepsis [71], investigators from the Radboud University Medical Center are currently recruiting an expected 200 participants for a new multi-center clinical trial with IFN-γ in patients with candidemia (NCT04979052, Table 2).

### 2.4. Cell Apoptosis

In addition to impaired production of effector molecules, sepsis patients of all age groups [7] present with severe apoptotic depletion of CD4+ and CD8+ T cells, B cells, and dendritic cells [68], which results in lymphopenia [2] and is associated with sepsis severity and mortality [3,4].

Interleukin-7 is a pluripotent cytokine, essential for T-cell survival and expansion [72]. Additionally, it has been found to modulate the effector to memory cell transition, as well as enhance immune reconstitution, diversify TCR repertoire [72], and restore delayed-type hypersensitivity (DTH) responses [73]. CYT107 is a recombinant human IL-7 (rhIL-7) produced by eukaryotic cells [72,73]. In a clinically relevant animal model of sepsis, CYT107 administration resulted in decreased CD4+ and CD8+ T-cell apoptosis [73]. Importantly, this study linked IL-7 administration in sepsis to improved survival. Similar results have been reported by different groups, including Rosenberg and colleagues, who described a dose-dependent increase in CD4+ and CD8+ lymphocyte numbers after rhIL-7 administration [74]. In addition to its antiapoptotic properties, CYT107 administration also induced T-cell proliferation and enhanced interferon-γ production, possibly contributing to the reported improvement in survival [73]. In the IRIS-7 trial (NCT02640807, Table 1), CYT107 administration caused a 3- to 4-fold increase in circulating CD4+ and CD8+ T cells, as well as in absolute lymphocyte count [35,75].

Likely due to the existence of a regulatory feedback loop [73], rhIL-7 therapy has continuously shown to be safe and well-tolerated [74,75,76]. Despite being closely related to IL-2, it rarely induces fever, capillary leak syndrome, or other manifestations of hyperinflammation [72,73]. In addition to its safety and tolerability, rhIL-7 therapy is also characterized by its long-lasting effects [74,76], and recent data suggest that complexing rhIL-7 to an anti-IL-7 monoclonal antibody can significantly increase its efficacy, likely due to a prolonged in vivo half-life [73,77].

Other cytokines have also been shown to possess immunorestorative properties, although none seem to be as potent as IL-7 [72]. For example, another γ-chain cytokine, interleukin-15, has shown promise in early studies of sepsis [14]. Like the closely related IL-7, IL-15 also augments the expression of antiapoptotic Bcl-2 and intensifies IFN-γ production [78], but seems to affect dendritic cells and NK cells more than CD4+ and CD8+ T cells [79]. In addition to modulating NK cell development and function, IL-15 displays a wide range of effects across both the innate and adaptive immune systems [72,78,79], and it has been linked to improved survival in CLP mice [80].

Another promising option that targets decreased cell counts is granulocyte–macrophage colony-stimulating factor. GM-CSF plays a role in emergency myelopoiesis, as it stimulates the production and differentiation of neutrophils, monocytes, macrophages, and myeloid-derived dendritic cells, as well as their antibacterial functions [2,65,81]. Because of its ability to prime immune cells for cytokine production and phagocytosis [22], thus enhancing host defenses, GM-CSF has been widely studied for the treatment of sepsis [82,83]. Its administration has shown clinical improvement in multiple studies [81,84] but no survival benefits. It is important to note, however, that given our increasing understanding of sepsis pathophysiology, different clinical endpoints such as long-term survival and functionality may be more clinically relevant than short-term mortality [82].

Importantly, past clinical studies of GM-CSF have integrated patient stratification in their protocols [85], often based on the expression of human leukocyte antigen-DR (HLA-DR) by monocytes (NCT02361528, Table 1) [36]. HLA-DR has recently emerged as a potential measure of the sum effect of pro- and anti-inflammatory mechanisms and may be able to identify the patient’s immune balance [22,86]. Its use in routine clinical context has practical limitations [87,88], but new quantification methods are currently the subject of extensive research [88,89], and this biomarker has already been used to stratify patients in clinical trials. It is currently considered the best marker of immunoparalysis in sepsis [22,86]. TNF-α response has also been used to guide treatment with GM-CSF in ongoing clinical trials (NCT03768844, NCT05266001, Table 2) [51,52].

### 2.5. Antigen Presentation

Dendritic cells are the ultimate antigen-presenting cells (APCs) and, once activated, are able to stimulate or suppress T-cell function [90,91]. In patients with sepsis, this population of cells undergoes extensive apoptosis, alterations in the cytokine profile, and reduced expression of HLA-DR, which is crucial for antigen-dependent responses [14,92].

Thymosin α1 (Tα1) is an endogenous lymphopoietic factor derived from the thymus [91], which has been used for the treatment of chronic viral infection and certain cancers and as a vaccine enhancer [90]. This thymic peptide is regarded as a promising immunoregulatory agent due to its ability to activate Toll-like receptors (TLRs) and DCs, enhance antigen presentation, and augment T-cell-mediated immune responses [91,93]. Through the modulation of different TLRs, Tα1 is able to balance pro- and anti-inflammatory mechanisms; not only can it stimulate the production of pro-inflammatory mediators such as IL-2 and interferons [93], but it can also increase the percentage of regulatory T cells and IL-10 production to ward off excessive inflammation [94]. As such, Tα1 therapy represents an encouraging way forward when it comes to managing the immune dysregulation seen in sepsis [94,95]. In a single-blinded, multi-center RCT, Tα1 administration to patients with sepsis decreased in-hospital mortality, as well as 28-day mortality [94]. Furthermore, it also improved mHLA-DR expression, which correlates with an improved immune response [94]. Similar results have been described by other authors: a systematic review of RCTs including 1354 patients also attributed survival benefits to Tα1 therapy, as well as improvement in clinical indicators such as APACHE II score and ICU days [94]. This therapeutic intervention has generally been considered safe and well-tolerated, with the most commonly observed side effects being local irritation and discomfort at the site of injection [93,94].

In the context of sepsis, thymosin α1 has also been studied in combination with ulinastatin (UTI). UTI is a trypsin inhibitor found in human urine with demonstrated anti-inflammatory and anti-apoptotic properties [96,97]. According to a recent meta-analysis, combination therapy may reduce 28-day and 90-day mortality in a dose-dependent manner [96]. The potential of UTI as a single agent in sepsis management is currently not well elucidated, and it is unclear whether Tα1, UTI, or the combination is responsible for the beneficial effects seen in existing studies [96,97]. Additional investigation is needed to provide conclusive evidence regarding the efficacy of these interventions, as well as dosage and treatment course considerations. Several clinical trials are currently ongoing (Table 2).

Another strategy for the augmentation of DC function is treatment with Fms-like tyrosine kinase-3 ligand (Flt3L). It has been dubbed as a DC growth factor, as it quickly prompts the expansion of dendritic cell subsets across different tissues [92,98]. In the context of sepsis, the Flt3 ligand has mostly been studied in animal models of thermal injury [99,100]. In this population, Flt3L treatment can augment the numbers of several immune cell types, boost cytokine production [99], restore the expression of MHC class II molecules and co-stimulatory signals, and ultimately increase survival [100]. Blockade of CD155 has also been shown to improve survival in septic mice and restore normal DC cytokine production [101].

### 2.6. Inhibitory Immune Checkpoints

In order to maintain homeostasis, leukocytes express negative co-stimulatory molecules, which inhibit signaling through the T-cell receptor (TCR) and CD28 [102]. Several of these immune checkpoints have been identified, such as CTLA-4, BTLA, LAG-3, and TIM-3, but the programmed cell death receptor-1 (PD-1) pathway remains by far the most studied in the context of sepsis. A postmortem study performed on patients who died of sepsis revealed upregulation of the PD-1 pathway on splenic T cells, and dendritic and epithelial cells from the lungs [17]. This increased expression of PD1 and its ligands, PD-L1 and PD-L2, was associated with a 90% decrease in cytokine production [103] and has been linked to the dysfunctional presentation of antigens, impaired humoral immunity, and decreased phagocytosis [102,104]. In fact, PD-L1 expression on monocytes is an independent predictor of 28-day mortality [102,105] and can be used to stratify patients’ risk levels and guide therapeutic decisions [106,107].

PD-1 and/or CTLA-4 modulation in melanoma, renal cell carcinoma, and non-small-cell lung cancer has been tremendously successful [7], and monoclonal antibodies that target these immune checkpoints are currently EMA and FDA approved [105]. These antibodies, broadly referred to as immune checkpoint inhibitors, have been linked to autoimmune reactions, with immune-mediated adverse events being reported in >3% of patients [29,107]. However, patients with sepsis would not require long-term treatment, and administration of such antibodies in two animal models of sepsis showed no unexpected adverse events [108]. Nivolumab is a fully human, EMA and FDA approved, anti-PD-1 monoclonal antibody that has shown benefit in the treatment of several advanced cancers, including melanoma, non-small-cell lung cancer, renal cell carcinoma, and malignant mesothelioma [109]. Hotchkiss and colleagues recently carried out a phase 1b clinical trial (NCT02960854, Table 1) aiming to study the safety, tolerability, and PK/PD of nivolumab in patients with sepsis, which revealed a progressive increase in mHLA-DR expression and no unexpected safety findings [107].

Like IL-7, anti-PD1 mAbs increase the production of IFN-γ and result in a net antiapoptotic effect [110]. Both seem to restore immune function through differing, although complementary, mechanisms and CLP mice that received combination therapy showed additive effects on lymphocyte proliferation, IFN-γ production, and CD28 expression [110]. Anti-PD-L1 mAb has also been shown to improve signaling through the IL-7 receptor, which further supports the rationale behind combination therapy in sepsis management [111].

Short-acting, low-molecular-weight peptides such as Compound 8 [112] and LD01 [113] have been proposed as an alternative to monoclonal antibodies, thanks to their lower immunogenicity and production costs. Another alternative is the modulation of immune checkpoint signaling pathways by existing drugs. Early administration of mycophenolate mofetil, an immunosuppressant used to prevent transplant rejection, has been shown to restrict PD-1 expression by regulatory T cells, decrease bacterial load, and alter cytokine production profiles in mice [114].

### 2.7. Cell Metabolism and Intracellular Signaling Pathways

Although anti-inflammatory strategies have not typically been successful in the treatment of sepsis, the discovery of new signaling pathways and molecular mechanisms has resulted in a newfound interest in this area. The novel ALK-EGFR-AKT pathway has recently been proposed as a therapeutic target for sepsis research [115]. During sepsis, ALK expression in monocytes and macrophages is upregulated, and genetic and pharmacological inhibition of ALK or STING have both corrected hyperinflammation and improved survival in mice [115,116]. LDK378, also known as ceritinib, is currently approved by the FDA and EMA for the treatment of metastatic non-small-cell lung cancer with ALK rearrangement [115]. This second-generation ALK inhibitor has been shown to reduce the release of pro-inflammatory cytokines such as TNF-α and IL-6 in CLP mice, which in turn improved microcirculation and decreased organ dysfunction [117]. According to Zeng and colleagues, treatment with ceritinib substantially protected mice against sepsis and lethal endotoxemia [115].

Sirtuin modulation has also emerged as a novel therapeutic strategy for the treatment of sepsis. These enzymes, in particular sirtuin 1 (SIRT1), are responsible for sensing the metabolic reprogramming of immune cells in the initial phase of sepsis [118]. On the one hand, SIRT1 inhibition has been shown to reverse endotoxin tolerance and re-shift metabolism back to glycolysis during the more immunosuppressive stage of the disease [2,118]. On the other hand, SIRT1 activator compounds, such as resveratrol or the synthetic SRT3025, have been shown to increase bacterial clearance and reduce inflammatory cytokines in CLP mice, improving survival [119] Given the ability of these therapies to modulate the immune response, it is imperative that the patient’s immune status is adequately characterized before treatment.

### 2.8. Gut Dysbiosis

A hallmark of critical inflammation [120], gut dysbiosis is often exacerbated by the supportive interventions used in sepsis management, such as broad-spectrum antibiotics and artificial nutrition [120,121]. Given its current success in recurrent *Clostridium difficile* infection, fecal microbiota transplant (FMT) has emerged as a possible therapeutic strategy in other disorders, such as inflammatory bowel disease and sepsis [122]. FMT therapy aims at re-establishing a normal gut microbiota through the introduction of feces from a healthy donor via nasogastric tube or colonoscopy [121]. Although experience with FMT in sepsis remains limited to case reports and animal studies, the strategy appears promising and seems particularly effective in gut-derived infections [122]. For example, in a 44-year-old woman with persistent sepsis and watery diarrhea following vagotomy, transplantation with fecal microbiota from a healthy, closely related donor led to the resolution of symptoms and restoration of the gut microbiome [123]. Other microbiota-directed therapies have been proposed, such as probiotics [120,120], but the results have not been consistent and despite the risk of additional inflammation and immunogenicity concerns [122], the preliminary FMT appears to be the most promising of the bunch. A randomized clinical trial studying FMT and/or probiotics in sepsis is currently ongoing (ChiCTR-INR-17011642) [56].

### 2.9. Cellular Therapies

Mesenchymal stem/stromal cells (MSCs) have potent immunomodulatory and tissue-regenerative abilities [124] and can alter their phenotype depending on the inflammatory environment [125], potentially restoring immune homeostasis. Present in a variety of tissues, they are easily harvested and possess low immunogenicity [125]. If activated in an inflammatory setting, MSCs develop an anti-inflammatory phenotype, MSC-2. Otherwise, they present with a pro-inflammatory phenotype known as MSC-1, which reduces immune cell apoptosis [124]. Although the effects of MSCs on the immune response and the different organ systems have been extensively reviewed elsewhere [124,126,127], the general consensus is that they act through cell-to-cell contact and the release of soluble factors and exosomes [125,128] to reduce bacterial burden, regulate cytokine homeostasis and ultimately, decrease organ dysfunction and short-term mortality [124,125]. In addition to their unique immunomodulatory properties, MSCs appear to act synergistically when combined with antibiotics [127].

Existing clinical trials mostly report MSC infusion as well tolerated and void of serious adverse effects [29,125]. However, some uncertainties remain. Due to the lack of standardized isolation and culture procedures, as well as the variability of MSC action, study results are often inconsistent or even conflicting [124]. There is also concern regarding thrombosis, anaphylactic shock, genetic instability, and malignant transformation [124,127]. In addition to these important clinical hurdles, the logistical barriers to real-life MSC use must be likewise considered. Industrial production of MSCs may result in cell products with slightly different properties than smaller-scale MSC production in academic centers, and these may even vary from one production facility to another [126]. MSC availability, such as other blood products, is heavily dependent on the donors [127], and since sepsis and septic shock are medical emergencies, a reserve supply of MSCs must be available. However, cryopreservation and storage may diminish the effectiveness of MSCs [126,127].

The genetic manipulation of T cells with a chimeric antigen receptor (CAR) to obtain T cells capable of identifying and targeting specific antigens without the need for antigen presentation has also garnered interest in the context of infectious diseases, though research remains extremely preliminary [129,130].

## 3. The COVID-19 Example: A Viral Sepsis

Even though the pathogens most frequently implicated in the etiology of sepsis are bacteria (and, to a lesser extent, fungi), sepsis can also occur due to viral infection [19]. Undoubtedly, the recent COVID-19 pandemic taught us a lot in this regard. Although most COVID-19 cases were mild or moderate, during the height of the pandemic, 15–20% of patients progressed to severe respiratory infection and adult respiratory distress syndrome (ARDS) [19], possibly resulting in septic shock or multiorgan failure [131]. Like in traditional sepsis, these patients presented with a dysregulated immune response, with hyperinflammation, activation of the coagulation cascade, and a cytokine storm, as well as lymphocyte exhaustion and the activation of immune checkpoints [19,131]. Although immune disruption is not as pronounced in COVID-19 as in traditional bacterial sepsis [18], the similarities are evident and severe COVID-19 should be regarded as viral sepsis [19].

Similarly to traditional sepsis, the host response to COVID-19 is extremely heterogeneous, and different patients might benefit from completely different treatment strategies [132]. The determination of disease severity, as well as the identification of immune-stratifying biomarkers, is imperative to adequately guide therapeutic decisions. In the early stages of the disease, eliminating or decreasing viral load is likely to limit the subsequent immune dysregulation, which validates the administration of antiviral agents such as remdesivir [132]. A similar approach is followed in bacterial sepsis, where early administration of antibiotics and appropriate source control interventions are key pillars of sepsis management. However, when the disease progresses and patients become critically ill, antimicrobial therapies do not seem to be as effective [18,133]. Once the immune response becomes unbalanced, immunotherapy comes into play, and patient stratification gains importance.

Changes in biomarkers such as C-reactive protein (CRP), ferritin, and soluble urokinase plasminogen activator receptor (suPAR) indicate worsening inflammation, and the administration of anti-inflammatory therapies should be considered [132]. Corticosteroids, for example, have proven to be highly effective at curbing the excessive inflammation in severe disease but would be detrimental to the natural immune response to the virus in earlier stages [134]. In addition to corticosteroids, critical COVID-19 patients with high serum levels of IL-6 also benefited from the administration of tocilizumab, a monoclonal antibody that functions as a competitive inhibitor of the IL-6 receptor [18]. Anakinra, an IL-1 receptor antagonist, has also been successful in the treatment of COVID-19 patients with lung hyperinflammation and elevated suPAR levels [18,132], a biomarker that indicates the activation of IL-1 signaling [135].

On the other hand, in patients who present with signs of immunoparalysis (illustrated by decreased monocytic HLA-DR, lymphopenia, and the presence of opportunistic infections), immunostimulatory therapies such as IFN-γ or even IL-7 become increasingly valid approaches [18,132]. Similarly to COVID-19, therapeutic decisions in sepsis should also be grounded in the adequate characterization of the patient’s current immune status.

## 4. Discussion and Conclusions

As traditional treatment protocols have evolved, in-hospital mortality from sepsis has substantially decreased [136], but those who survive the hyperinflammation and subsequent organ damage report reduced quality of life and often do not survive the long-term complications such as secondary infection. Currently, no sepsis-specific therapies exist, and research in this area is well known for having highly promising results in animal studies, which fail at a clinical level. These therapies, which include immune checkpoint inhibitors, cytokines, and growth factors, are frequently studied at a pre-clinical level without the inclusion of standard-of-care practices, such as antimicrobial therapy and other supportive measures. Furthermore, rather than the commonly used short-term mortality, different endpoints such as long-term survival and functionality may be more clinically relevant.

In sepsis, the immune response is highly heterogeneous, and the employed therapeutic strategies must address both the hyperinflammation and the immunosuppression—stratifying patients according to their overall immune balance is imperative. Furthermore, in addition to diagnostic and prognostic biomarkers, there is a pressing need for the development of predictors of therapeutic efficacy. There is still a long way to go, but important strides have been made: decreased monocytic HLA-DR and increased levels of circulating IL-10 have shown potential for stratification for GM-CSF or IFN-γ treatments, and T-cell counts (CD4+ and Treg) may be helpful in stratifying for IL-7 therapy [92]. The ratio of serum IFN-γ to IL-10 has been proposed as a potential biomarker to guide corticosteroid therapy [137], and during the COVID-19 pandemic, serum IL-6 was used to guide for the administration of immunosuppressive treatment [138]. Biologically active adrenomedullin (BioADM) is currently regarded as a biomarker of cardiovascular and endothelial status, and it can successfully monitor the evolution of septic shock and the success of the utilized therapies. Adrecizumab, a non-neutralizing anti-adrenomedullin antibody, has shown promising results in animal studies and a phase II clinical trial (NCT03085758, Table 1) [46,138].

The host response during sepsis is an extremely complex and non-linear process, which can result in emergent behavior that cannot be captured by single time points and isolated analyses of specific host response features [139]. This abnormal behavior of the immune system involves the interplay between immune cells, cytokines, the coagulation cascade, the endothelial response, the complement system, the gut microbiome, the neuroendocrine system, altered energy metabolism, the failure of whole-organ systems, mechanical and pharmacological interventions by doctors, the erosive sequelae of comorbidities, one or more causative pathogens, and other factors [140]. Therefore, a single pharmacodynamic approach will probably be unsuccessful in tackling the widespread dysregulation of the inflammatory response that occurs during septic shock. Although there are pathophysiological differences between early inflammatory and later immunosuppressive stages, both lead to a high risk of mortality. Consequently, clinical studies employing a combination of therapeutic interventions in each of these phases should add significant value in the improvement of clinical outcomes [141]; drug combinations such as thymosion α1 and ulinastatin, or interleukin-7 plus anti-PD-1 monoclonal antibodies, appear to be the best path forward. The existence of redundant biological pathways also makes it difficult for single-target therapy to achieve satisfactory results [142]. To this effect, multi-target agents such as heparan sulfate octadecasaccharide (18-mer) have very recently emerged as potential new strategies for the management of sepsis [143]. Cellular therapies, such as mesenchymal stem cells, have also garnered interest due to their ability to adapt according to the present inflammatory environment. However, the heterogeneity observed in sepsis patients and in stem cell products might lead to a disconnect between clinical and pre-clinical studies, hindering clinical translation [126]. Moreover, the applicability of advanced therapies in a real-life setting remains constrained by logistical hurdles that are aggravated by the high prevalence of sepsis and septic shock [127].

Future drug development must be tailored to the patient, and additional, high-quality data are still needed to provide conclusive evidence regarding the efficacy of most of the therapeutic strategies discussed in this review. Additionally, researchers should look at therapeutic advances in other areas of medicine—for example, the success of checkpoint inhibitors in oncology has majorly contributed to sepsis research. Similarly, the recent COVID-19 pandemic created a one-of-a-kind situation, with unprecedented efforts in research and a large number of drugs being evaluated in a very brief amount of time [18]. The repurposing of existing drugs was highly investigated, and therapies used to slow down the hyperinflammation in patients with severe COVID-19 were extremely successful, such as several trials associated mortality benefits with corticosteroids and cytokine blockers such as tocilizumab (IL-6R blocker) and anakinra (IL-1 inhibitor) [18,19]. Although anti-inflammatory strategies have generally been unsuccessful in traditional sepsis and septic shock, there are definitely lessons to be learned from severe COVID-19, which is essentially sepsis caused by viral infection [131,144].

As shown in Table 2, current ongoing clinical trials largely seem to focus on augmenting the immune response, but a personalized approach is urgently needed. Critically ill patients included in sepsis clinical trials should be divided and stratified according to sepsis severity and different pathophysiological phenotypes (achieved by biomarker identification) in an attempt to maximize the signal-to-noise ratio of the investigational medical products being tested. Although it is unlikely that a “magic bullet” approach will be efficacious in sepsis as it is classified nowadays, the data analyzed in this manuscript suggest that a subgroup classification of patients (based on biomarkers, stage of disease, severity) may demonstrate to be crucial in identifying a more patient-specific treatment, leading to improved clinical outcomes. The ImmunoSep trial (NCT04990232, Table 2) is a pioneer international, double-blind, phase 2, randomized clinical trial on personalized immunotherapy in sepsis, where patients are randomized according to their immune characteristics [145]. Enrolled patients are stratified according to their ferritin and monocytic HLA-DR levels and subsequently allocated to placebo or active immunotherapy; in addition to standard-of-care treatment, patients with high ferritin (fulminant hyperinflammation) will receive intravenous anakinra and patients with low mHLA-DR (sepsis-induced immunoparalysis) will receive subcutaneous IFNγ [135]. Although challenges such as the screening failure rate are expected, this is the first study applying precision medicine concepts to immunotherapy for sepsis. This innovative project is funded by the European Union’s Horizon 2020 research and innovation program, and even though the trial is not powered to study mortality, it ultimately aims to integrate novel immunotherapeutic approaches in routine clinical practice [135].

## Figures and Tables

**Table 1:** Completed interventional studies for the treatment of sepsis and septic shock from the last 10 years.

Title	ClinicalTrials.gov Identifier	Intervention	Phase (Participants)	Study Design	Primary Outcome	Study Start Date	Study Progress	Primary Sponsor	Ref.
Studying Complement Inhibition in Early, Newly Developing Septic Organ Dysfunction	NCT02246595	CaCP29	Phase 2 (n = 72)	Quadruple-blinded, randomized, parallel assignment, placebo-controlled trial	1. Plasma concentration of CaCP29 2. Pharmacodynamic effects of CaCP29 on the change from baseline in plasma concentrations of C5a 3. Safety variables	April 2014	Completed, No Results Posted	InflaRx GmbH	[31]
In Vivo Effects of *C*1-esterase Inhibitor on the Innate Immune Response During Human Endotoxemia	NCT01766414	C1-esterase inhibitor	Phase 3 (n = 20)	Triple-blinded, randomized, parallel assignment, placebo-controlled trial (unspecified blinding)	Neutrophil phenotype and redistribution	September 2013	Completed, No Results Posted	Radboud University Medical Center	[32]
Vorapaxar in the Human Endotoxemia Model	NCT02875028	Vorapaxar	Phase 4 (n = 16)	Quadruple-blinded, randomized, crossover assignment, placebo-controlled trial	Changes in Prothrombin Fragments F1+2	June 2016	Completed	Medical University of Vienna	[33]
A Trial of Validation and Restoration of Immune Dysfunction in Severe Infections and Sepsis	NCT03332225	Anakinra; Recombinant human interferon-γ	Phase 2 (n = 36)	Quadruple-blinded, randomized, parallel assignment, placebo-controlled trial	28-day mortality	December 2017	Completed, No Results Posted	Hellenic Institute for the Study of Sepsis	[34]
A Study of IL-7 to Restore Absolute Lymphocyte Counts in Sepsis Patients	NCT02640807	CYT107: Interleukin-7	Phase 2 (n = 27)	Quadruple-blinded, randomized, parallel assignment, placebo-controlled trial	Immune reconstitution of lymphocytopenic sepsis patients	January 2016	Completed	Revimmune	[35]
GM-CSF to Decrease ICU Acquired Infections	NCT02361528	GM-CSF	Phase 3 (n = 166)	Quadruple-blinded, randomized, parallel assignment, placebo-controlled trial	Number of patients presenting at least one ICU-acquired infection at D28 or ICU discharge	September 2015	Completed, No Results Posted	Hospices Civilis de Lyon	[36]
Efficacy of Thymosin Alpha 1 on Improving Monocyte Function in Sepsis	NCT02883595	Thymosin Alpha 1	Phase 4 (n = 20)	Quadruple-blinded, randomized, parallel assignment, placebo-controlled trial	Flow cytometric measuring of phagocytosis (CD11b, CD64), antigen presenting (HLA-DR, CD86, and PD-L1), and apoptosis (active caspase 3) on monocytes	March 2016	Completed, No Results Posted	Sun Yat-sen University	[37]
The Efficacy and Safety of Tα1 for Sepsis	NCT02867267	Thymosin Alpha 1	Phase 3 (n = 1106)	Quadruple-blinded, randomized, parallel assignment, placebo-controlled trial	28-day all-cause mortality	September 2016	Completed, No Results Posted	Sun Yat-sem University	[38]
Effects of shengmai injection combined with thymosin on cellular immune function in patients with sepsis and low immune function: a prospective, randomized, controlled trial	N/A (ChiCTR identifier: ChiCTR2100043911)	Shengmai injection; Thymosin injection	N/A (n = 90)	Parallel assignment, randomized, placebo-controlled trial	Peripheral blood T-cell subsets	January 2019	Completed	The Ninth People’s Hospital of Suzhou	[39]
Ulinastatin Treatment in Adult Patients with Sepsis and Septic Shock in China	NCT02647554	Ulinastatin	Phase 4 (n = 347)	Quadruple-blinded, randomized, parallel assignment, placebo-controlled trial	28-day all-cause mortality	December 2016	Completed, No Results Posted	Peking Union Medical College Hospital	[40]
A Study of Nivolumab Safety and Pharmacokinetics in Patients with Severe Sepsis or Septic Shock	NCT02960854	Nivolumab	Phase 1 (n = 38)	Double-blinded, randomized, parallel assignment, placebo-controlled trial	1. Percentage of Incidence Rates of Serious Adverse Events (SAEs), Adverse Events (AEs), Immune-mediated AEs, AEs Leading to Discontinuation, and Deaths 2. Composite of Vital Signs and Electrocardiogram 3. Peak Nivolumab Serum Concentration 4. Trough Nivolumab Serum Concentration 5. Average Nivolumab Serum Concentration 6. Time of Maximum Observed Concentration 7. Area Under the Serum Concentration–time Curve From Time Zero to Time of Last Quantifiable Concentration 8. Total Clearance 9. Volume of Distribution 10. Half-life	December 2016	Completed	Bristol-Myers Squibb	[41]
Effect of Mesenchymal Stromal Cells on Sepsis and Sepsis and Septic Shock	NCT05283317	Mesenchymal Stem Cells	Phase 1, Phase 2 (n = 30)	Single-blinded, non-randomized, parallel assignment interventional trial	28-day mortality	March 2018	Completed, No Results Posted	TC Enciyes University	[42]
Randomized, Parallel Group, Placebo Control, Unicentric, Interventional Study to Assess the Effect of Expanded Human Allogeneic Adipose-derived Mesenchymal Adult Stem Cells on the Human Response to Lipopolysaccharide in Human Volunteers	NCT02328612	Intravenous infusion of cells	Phase 1 (n = 32)	Randomized, parallel assignment, open-label trial	Inflammatory response as measured by laboratory measurements and functional assays of innate immunology	October 2014	Completed, No Results Posted	Tigenix S.A.U.	[43]
Cellular Immunotherapy for Septic Shock: A Phase I Trial	NCT02421484	Allogeneic Mesenchymal Stromal Cells	Phase 1 (n = 9)	Single-group assignment, open-label trial	Number of adverse events as a measure of safety and tolerability	May 2015	Completed, No Results Posted	Ottawa Hospital Research Institute	[44]
Pharmacokinetics of XueBiJing in Patients with Sepsis	NCT03475732	XueBiJing injection	N/A (n = 35)	Single-group assignment, open-label trial	Plasma concentrations of XueBiJing injection compounds	March 2018	Completed, No Results Posted	Southeast University, China	[45]
Treatment of Patients with Early Septic Shock and Bio-Adrenomedullin (ADM) Concentration > 70 pg/mL with ADRECIZUMAB	NCT03085758	Adrecizumab	Phase 2 (n = 301)	Double-blinded, randomized, parallel assignment, placebo-controlled trial	1. 90-day mortality 2. Interruption of infusion due to intolerability of adrecizumab 3. Number of participants with treatment-emergent adverse events per treatment group 4. Number of participants with treatment-emergent adverse events per treatment group with mild severity treatment-emergent events 5. Number of participants with treatment-emergent adverse events per treatment group with moderate severity treatment-emergent events 6. Number of participants with treatment-emergent adverse events per treatment group with severe severity treatment-emergent events	December 2017	Completed	Adrenomed AG	[46]
Effects of Microcirculation of IgGAM in Severe Septic/Septic Shock Patients	NCT02655133	Pentaglobin^®^	Phase 2 (n = 20)	Quadruple-blinded, randomized, parallel assignment, placebo-controlled trial	Perfused vessel density (PVD)	January 2016	Completed, No Results Posted	Università Politecnica delle Manche	[47]
Efficacy of Mw Vaccine in Treatment of Severe Sepsis	NCT02025660	Mw	Phase 2, Phase 3 (n = 50)	Double-blinded, randomized, parallel assignment, placebo-controlled trial	4-week mortality	August 2013	Completed, No Results Posted	Postgraduate Institute of Medical Education and Research	[48]
Safety, Tolerability, Pharmacokinetics and Pharmacodynamics of 3 Doses of MOTREM in Patients with Septic Shock	NCT03158948	MOTREM: Nangibotide	Phase 2 (n = 50)	Quadruple-blinded, randomized, parallel assignment, placebo-controlled trial	1. Vital signs 2. ECG 3. Number of patients with clinically relevant abnormal laboratory values 4. Presence of anti-LR12 antibodies 5. Adverse events	July 2017	Completed, Results Submitted	Inotrem	[49]

**Table 2:** Ongoing interventional studies for the treatment of sepsis and septic shock from the last 10 years.

Title	ClinicalTrials.gov Identifier	Intervention	Phase	Study Design	Primary Outcome	Study Start Date	Study Progress	Primary Sponsor	Ref.
Safety and Efficacy of Interferon-gamma 1β in Patients with Candidemia	NCT04979052	Interferon Gamma-1β	Phase 2 (200 estimated participants)	Randomized, parallel assignment, open-label adaptive trial	Time to first negative blood culture	March 2022	Recruiting	Redboud University Medical Center	[50]
GM-CSF for Reversal of Immunoparalysis in Pediatric Sepsis-induced MODS Study	NCT03769844	GM-CSF	Phase 4 (120 estimated participants)	Non-randomized, sequential assignment, open-label trial	TNF-α response	December 2018	Active, not recruiting	Nationwide Children’s Hospital	[51]
GM-CSF for Reversal of Immunoparalysis in Pediatric Sepsis-induced MODS Study 2	NCT05266001	GM-CSF	Phase 3 (400 estimated participants)	Quadruple-blinded, randomized, parallel assignment, placebo-controlled trial	Cumulative 28-day pediatric logistic organ dysfunction (PELOD)-2 score	June 2022	Recruiting	Nationwide Children’s Hospital	[52]
A prospective, double-blind, randomized controlled trial study of the effect of immune regulation on the prognosis of sepsis	N/A (ChiCTR identifier: ChiCTR2200060069)	Thymopentin	Phase 4 (426 estimated participants)	Double-blinded, randomized, parallel assignment, placebo-controlled trial	28-day mortality rate	June 2022	Not yet recruiting	The First Affiliated Hospital with Nanjing Medical University	[53]
Application of Immune Cell-oriented Clinical Phenotypic Guides the Treatment of Sepsis	N/A (ChiCTR identifier: ChiCTR2100048326)	Methylprednisolone; Thymosin α1	N/A (200 estimated participants)	Parallel assignment randomized trial (blinding unspecified)	28-day patient mortality rate	July 2021	Not yet recruiting	Renji Hospital, Shanghai Jiaotong University School of Medicine	[54]
Clinical Efficacy of Ulinastatin for Treatment of Sepsis with Systemic Inflammatory Response Syndrome	NCT05391789	Ulinastatin	Phase 3 (120 estimated participants)	Triple-blinded, randomized, parallel assignment, placebo-controlled trial	ΔSOFA	July 2022	Not yet recruiting	Huashan Hospital	[55]
Clinical research of fecal microbiota transplantation and probiotics regulating intestinal flora diversity on the systemic immune function in septic patients	N/A (ChiCTR identifier: ChiCTR-INR-17011642)	Fecal microbiota transplantation; Probiotic	N/A (80 estimated participants)	Parallel assignment, randomized trial (blinding unspecified)	1. Gut microbiota composition 2. Immunoglobulin 3. Lymphocyte immune analysis	July 2017	Not yet recruiting	Chinese food fermentation industry research institute	[56]
Advanced Mesenchymal Enhanced Cell Therapy for Septic Patients	NCT04961658	GEM00220: Enhanced MSCs	Phase 1 (21 estimated participants)	Sequential assignment, non-randomized, open-label, dose-escalation trial	1. Adverse Events 2. Maximum Feasible Tolerated Dose	August 2021	Recruiting	Northern Therapeutics	[57]
Personalized Immunotherapy in Sepsis	NCT04990232	Anakinra; Recombinant human IFNγ	Phase 2 (280 estimated participants)	Quadruple-blinded, randomized, parallel assignment, double-placebo-controlled trial	Mean total Sequential Organ Failure Assessment score	July 2021	Recruiting	Hellenic Institute for the Study of Sepsis	[58]
Efficacy and Safety of Therapy with IgM-enriched Immunoglobulin with a Personalized Dose vs. Standard Dose in Patients with Septic Shock	NCT04182737	IgM-enriched polyclonal immunoglobulins	Phase 3 (356 estimated participants)	Single-blinded, randomized, parallel assignment	All-cause, 28-day mortality	May 2020	Recruiting	Massimo Girandis	[59]
Efficacy, Safety and Tolerability of Nangibotide in Patients with Septic Shock	NCT04055909	Nangibotide	Phase 2 (355 estimated participants)	Quadruple-blinded, randomized, parallel assignment, placebo-controlled trial	Sequential organ failure assessment (SOFA) score	November 2019	Active, not recruiting	Inotrem	[60]

## Data Availability

Data is contained within the article.

## References

[1] Singer M., Deutschman C.S., Seymour C.W., Shankar-Hari M., Annane D., Bauer M., Bellomo R., Bernard G.R., Chiche J.-D., Coopersmith C.M. (2016). The Third International Consensus Definitions for Sepsis and Septic Shock (Sepsis-3). JAMA.

[2] Venet F., Monneret G. (2018). Advances in the Understanding and Treatment of Sepsis-Induced Immunosuppression. Nat. Rev. Nephrol..

[3] Hotchkiss R.S., Moldawer L.L., Opal S.M., Reinhart K., Turnbull I.R., Vincent J.-L. (2017). Sepsis and Septic Shock. Physiol. Behav..

[4] Adegboro B.A., Imran J., Abayomi S.A., Sanni E.O., Biliaminu S.A. (2021). Recent Advances in the Pathophysiology and Management of Sepsis: A Review. Afr. J. Clin. Exp. Microbiol..

[5] Gilbert J.A. (2018). Sepsis Care Bundles: A Work in Progress. Lancet Respir. Med..

[6] Rudd K.E., Johnson S.C., Agesa K.M., Shackelford K.A., Tsoi D., Kievlan D.R., Colombara D.V., Ikuta K.S., Kissoon N., Finfer S. (2020). Global, Regional, and National Sepsis Incidence and Mortality, 1990–2017: Analysis for the Global Burden of Disease Study. Lancet.

[7] Hotchkiss R.S., Monneret G., Payen D. (2013). Immunosupression in Sepsis: A Novel Understanding of the Disorder and a New Therapeutic Approach. Lancet Infect Dis..

[8] Rahmel T., Schmitz S., Nowak H., Schepanek K., Bergmann L., Halberstadt P., Hörter S., Peters J., Adamzik M. (2020). Long-Term Mortality and Outcome in Hospital Survivors of Septic Shock, Sepsis, and Severe Infections: The Importance of Aftercare. PLoS ONE.

[9] Prescott H.C., Costa D.K. (2018). Improving Long-Term Outcomes After Sepsis. Crit. Care Clin..

[10] Evans L., Rhodes A., Alhazzani W., Antonelli M., Coopersmith C.M., French C., Machado F.R., Mcintyre L., Ostermann M., Prescott H.C. (2021). Surviving Sepsis Campaign: International Guidelines for Management of Sepsis and Septic Shock 2021. Intensive Care Med..

[11] Barichello T., Generoso J.S., Collodel A., Petronilho F., Dal-Pizzol F. (2021). The Blood-Brain Barrier Dysfunction in Sepsis. Tissue Barriers.

[12] Mostel Z., Perl A., Marck M., Mehdi S.F., Lowell B., Bathija S., Santosh R., Pavlov V.A., Chavan S.S., Roth J. (2019). Post-Sepsis Syndrome- A n Evolving Entity That Afflicts Survivors of Sepsis. Mol. Med..

[13] Prescott H.C., Angus D.C. (2018). Enhancing Recovery From Sepsis. JAMA.

[14] Delano M.J., Ward P.A. (2016). Sepsis-Induced Immune Dysfunction: Can Immune Therapies Reduce Mortality?. J. Clin. Investig..

[15] Martin G.S., Mannino D.M., Moss M. (2006). The Effect of Age on the Development and Outcome of Adult Sepsis. Crit. Care Med..

[16] Weis S., Carlos A.R., Moita M.R., Singh S., Blankenhaus B., Cardoso S., Larsen R., Rebelo S., Schäuble S., Del Barrio L. (2017). Metabolic Adaptation Establishes Disease Tolerance to Sepsis. Cell.

[17] Boomer J.S., To K., Chang K.C., Takasu O., Osborne D.F., Walton A.H., Bricker T.L., Jarman S.D., Kreisel D., Krupnick A.S. (2011). Immunosuppression in Patients Who Die of Sepsis and Multiple Organ Failure. JAMA.

[18] Rondovic G., Djordjevic D., Udovicic I., Stanojevic I., Zeba S., Abazovic T., Vojvodic D., Abazovic D., Khan W., Surbatovic M. (2022). From Cytokine Storm to Cytokine Breeze: Did Lessons Learned from Immunopathogenesis Improve Immunomodulatory Treatment of Moderate-to-Severe COVID-19?. Biomedicines.

[19] Tufan Z.K., Kayaaslan B., Mer M. (2021). COVID-19 and Sepsis. Turk. J. Med. Sci..

[20] Lilly Announces Withdrawal of Xigris® Following Recent Clinical Trial Results. https://investor.lilly.com/news-releases/news-release-details/lilly-announces-withdrawal-xigrisr-following-recent-clinical.

[21] Lai P.S., Thompson B.T. (2013). Why Activated Protein C Was Not Successful in Severe Sepsis and Septic Shock: Are We Still Tilting at Windmills?. Curr. Infect. Dis. Rep..

[22] Davies R., O’Dea K., Gordon A. (2018). Immune Therapy in Sepsis: Are We Ready to Try Again?. J. Intensive Care Soc..

[23] Davenport E.E., Burnham K.L., Radhakrishnan J., Humburg P., Hutton P., Mills T.C., Rautanen A., Gordon A.C., Garrard C., Hill A.V.S. (2016). Genomic Landscape of the Individual Host Response and Outcomes in Sepsis: A Prospective Cohort Study. Lancet Respir. Med..

[24] Wang Y., Gloss B., Tang B., Dervish S., Santner-Nanan B., Whitehead C., Masters K., Skarratt K., Teoh S., Schibeci S. (2021). Immunophenotyping of Peripheral Blood Mononuclear Cells in Septic Shock Patients With High-Dimensional Flow Cytometry Analysis Reveals Two Subgroups With Differential Responses to Immunostimulant Drugs. Front. Immunol..

[25] Wang C., Liang G., Shen J., Kong H., Wu D., Huang J., Li X. (2021). Long Non-Coding RNAs as Biomarkers and Therapeutic Targets in Sepsis. Front. Immunol..

[26] Pierrakos C., Velissaris D., Bisdorff M., Marshall J., Vincent J.-L. (2020). Biomarkers of Sepsis: Time for a Reappraisal. Crit. Care.

[27] Munn Z., Peters M.D.J., Stern C., Tufanaru C., McArthur A., Aromataris E. (2018). Systematic Review or Scoping Review? Guidance for Authors When Choosing between a Systematic or Scoping Review Approach. BMC Med. Res. Methodol..

[28] Westphaln K.K., Regoeczi W., Masotya M., Vazquez-Westphaln B., Lounsbury K., McDavid L., Lee H., Johnson J., Ronis S.D. (2021). From Arksey and O’Malley and Beyond: Customizations to Enhance a Team-Based, Mixed Approach to Scoping Review Methodology. MethodsX.

[29] Denstaedt S.J., Singer B.H., Standiford T.J. (2018). Sepsis and Nosocomial Infection: Patient Characteristics, Mechanisms, and Modulation. Front. Immunol..

[30] Mira J.C., Brakenridge S.C., Moldawer L.L., Moore F.A. (2017). Persistent Inflammation, Immunosuppression and Catabolism Syndrome. Crit. Care Clin..

[31] ClinicalTrials.gov Studying Complement Inhibition in Early, Newly Developing Septic Organ Dysfunction (SCIENS) (NCT02246595). NCT02246595.

[32] ClinicalTrials.gov In Vivo Effects of C1-Esterease Inhibitor on the Innate Immune Response During Human Endotoxemia (NCT01766414). NCT01766414.

[33] ClinicalTrials.gov Vorapaxar in the Human Endotoxemia Model (NCT02875028). NCT02875028.

[34] ClinicalTrials.gov A Trial of Validation and Restoration of Immune Dysfunction in Severe Infections and Sepsis (NCT03332225). NCT03332225.

[35] ClinicalTrials.gov A Study of IL-7 to Restore Absolute Lymphocyte Counts in Sepsis Patients (NCT02640807). NCT02640807.

[36] ClinicalTrials.gov GM-CSF to Decrease ICU Acquired Infections (GRID) (NCT02361528). NCT02361528.

[37] ClinicalTrials.gov Efficacy of Thymosin Alpha 1 on Improving Monocyte Function in Sepsis (NCT02883595). NCT02883595.

[38] ClinicalTrials.gov The Efficacy and Safety of Ta1 for Sepsis (NCT02867267). NCT02867267.

[39] International Clinical Trials Registry Platform Effects of Shengmai Injection Combined with Thymosin on Cellular Immune Function in Patients with Sepsis and Low Immune Function: A Prospective, Randomized, Controlled Trial (ChiCTR2100043911). https://trialsearch.who.int/Trial2.aspx?TrialID=ChiCTR2100043911.

[40] ClinicalTrials.gov Ulinastatin Treatment in Adult Patients With Sepsis and Septic Shock in China (NCT02647554). NCT02647554.

[41] ClinicalTrials.gov A Study of Nivolumab Safety and Pharmacokinetics in Patients With Severe Sepsis or Septic Shock (NCT02960854). NCT02960854.

[42] ClinicalTrials.gov Effect of Mesenchymal Stromal Cells on Sepsis and Sepsis and Septic Shock (NCT05283317). NCT05283317.

[43] ClinicalTrials.gov Randomized, Parallel Group, Placebo Control, Unicentric, Interventional Study to Assess the Effect of Expanded Human Allogeneic Adipose-Derived Mesenchymal Adult Stem Cells on the Human Response to Lipopolysaccharyde in Human Volunteers (NCT02328612). NCT02328612.

[44] ClinicalTrials.gov Cellular Immunotherapy for Septic Shock: A Phase I Trial (NCT02421484). NCT02421484.

[45] ClinicalTrials.gov Pharmacokinetics of XueBiJing in Patients With Sepsis (NCT03475732). NCT03475732.

[46] ClinicalTrials.gov Treatment of Patients With Early Septic Shock and Bio-Adrenomedullin(ADM) Concentration > 70 Pg/Ml With ADRECIZUMAB (NCT03085758). NCT03085758.

[47] ClinicalTrials.gov Effects on Microcirculation of IgGAM in Severe Septic/Septic Shock Patients. https://clinicaltrials.gov/ct2/show/NCT02655133?id=NCT02655133+OR+NCT03475732+OR+NCT02025660+OR+NCT04182737+OR+NCT03013322+OR+NCT03085758+OR+NCT02442440+OR+NCT05469347+OR+NCT04123444+OR+NCT04055909&draw=2&rank=8&load=cart.

[48] ClinicalTrials.gov Efficacy of Mw Vaccine in Treatment of Severe Sepsis (NCT02025660). NCT02025660.

[49] ClinicalTrials.gov Safety, Tolerability, Pharmacokinetics and Pharmacodynamics of 3 Doses of MOTREM in Patients With Septic Shock (NCT03158948). NCT03158948.

[50] ClinicalTrials.gov Safety and Efficacy of Interferon-Gamma 1b in Patients with Candidemia (NCT04979052). NCT04979052.

[51] ClinicalTrials.gov GM-CSF for Reversal of Immunoparalysis in Pediatric Sepsis-Induced MODS Study (NCT03769844). NCT03769844.

[52] ClinicalTrials.gov GM-CSF for Reversal of Immunoparalysis in Pediatric Sepsis-Induced MODS Study 2 (NCT05266001). NCT05266001.

[53] International Clinical Trials Registry Platform A Prospective, Double-Blind, Randomized Controlled Trial Study of the Effect of Immune Modulation on the Prognosis of Sepsis (ChiCTR2200060069). https://trialsearch.who.int/Trial2.aspx?TrialID=ChiCTR2200060069.

[54] International Clinical Trials Registry Platform Application of Immune Cell-Oriented Clinical Phenotypic Guides the Treatment of Sepsis (ChiCTR2100048326). https://trialsearch.who.int/Trial2.aspx?TrialID=ChiCTR2100048326.

[55] ClinicalTrials.gov Clinical Efficacy of Ulinastatin for Treatment of Sepsis With Systemic Inflammatory Response Syndrome (NCT05391789). NCT05391789.

[56] International Clinical Trials Registry Platform Clinical Research of Fecal Microbiota Transplantation and Probiotics Regulating Intestinal Flora Diversity on the Systemic Immune Function in Septic Patients (ChiCTR-INR-17011642). https://trialsearch.who.int/Trial2.aspx?TrialID=ChiCTR-INR-17011642.

[57] ClinicalTrials.gov Advanced Mesenchymal Enhanced Cell Therapy for Septic Patients (NCT04961658). NCT04961658.

[58] ClinicalTrials.gov Personalized Immunotherapy in Sepsis (NCT04990232). NCT04990232.

[59] ClinicalTrials.gov Efficacy and Safety of Therapy With IgM-Enriched Immunoglobulin With a Personalized Dose vs Standard Dose in Patients With Septic Shock (NCT04182737). NCT04182737.

[60] ClinicalTrials.gov Efficacy, Safety and Tolerability of Nangibotide in Patients With Septic Shock (NCT04055909). NCT04055909.

[61] Guo R.F., Ward P.A. (2005). Role of C5a in Inflammatory Responses. Annu. Rev. Immunol..

[62] Ward P.A., Guo R.F., Riedemann N.C. (2012). Manipulation of the Complement System for Benefit in Sepsis. Crit. Care Res. Pract..

[63] Fattahi F., Zetoune F.S., Ward P.A. (2020). Complement as a Major Inducer of Harmful Events in Infectious Sepsis. Shock.

[64] ClinicalTrials.gov Clinical Assessment of Safety and Tolerability of the New Monoclonal Humanized Antibody CaCP29 (NCT01319903). NCT01319903.

[65] Van Der Poll T., Van De Veerdonk F.L., Scicluna B.P., Netea M.G. (2017). The Immunopathology of Sepsis and Potential Therapeutic Targets. Nat. Rev. Immunol..

[66] Silasi-Mansat R., Zhu H., Popescu N.I., Peer G., Sfyroera G., Magotti P., Ivanciu L., Lupu C., Mollnes T.E., Taylor F.B. (2010). Complement Inhibition Decreases the Procoagulant Response and Confers Organ Protection in a Baboon Model of Escherichia Coli Sepsis. Blood.

[67] Schoergenhofer C., Schwameis M., Gelbenegger G., Buchtele N., Thaler B., Mussbacher M., Schabbauer G., Wojta J., Jilma-Stohlawetz P., Jilma B. (2018). Inhibition of Protease-Activated Receptor (PAR1) Reduces Activation of the Endothelium, Coagulation, Fibrinolysis and Inflammation during Human Endotoxemia. Thromb. Haemost..

[68] Chiche L., Forel J.M., Thomas G., Farnarier C., Cognet C., Guervilly C., Zandotti C., Vély F., Roch A., Vivier E. (2012). Interferon-γ Production by Natural Killer Cells and Cytomegalovirus in Critically Ill Patients. Crit. Care Med..

[69] Leentjens J., Kox M., Koch R.M., Preijers F., Joosten L.A.B., Van Der Hoeven J.G., Netea M.G., Pickkers P. (2012). Reversal of Immunoparalysis in Humans in Vivo: A Double-Blind, Placebo-Controlled, Randomized Pilot Study. Am. J. Respir. Crit. Care Med..

[70] Döcke W.D., Randow F., Syrbe U., Krausch D., Asadullah K., Reinke P., Volk H.D., Kox W. (1997). Monocyte Deactivation in Septic Patients: Restoration by IFN-Gamma Treatment. Nat. Med..

[71] ClinicalTrials.gov The Effects of Interferon-Gamma on Sepsis-Induced Immunoparalysis (NCT01649921). NCT01649921.

[72] Mackall C.L., Fry T.J., Gress R.E. (2011). Harnessing the Biology of IL-7 for Therapeutic Application. Nat. Rev. Immunol..

[73] Unsinger J., McGlynn M., Kasten K.R., Hoekzema A.S., Watanabe E., Muenzer J.T., McDonough J.S., Tschoep J., Ferguson T.A., McDunn J.E. (2010). IL-7 Promotes T Cell Viability, Trafficking, and Functionality and Improves Survival in Sepsis. J. Immunol..

[74] Rosenberg S.A., Sportès C., Ahmadzadeh M., Fry T.J., Ngo L.T., Schwarz S.L., Stetler-Stevenson M., Morton K.E., Mavroukakis S.A., Morre M. (2006). IL-7 Administration to Humans Leads to Expansion of CD8+ and CD4+ Cells but a Relative Decrease of CD4+ T-Regulatory Cells. J. Immunother..

[75] Francois B., Jeannet R., Daix T., Walton A.H., Shotwell M.S., Unsinger J., Monneret G., Rimmelé T., Blood T., Morre M. (2018). Interleukin-7 Restores Lymphocytes in Septic Shock: The IRIS-7 Randomized Clinical Trial. JCI Insight.

[76] Lundström W., Fewkes N.M., Mackall C.L. (2012). IL-7 in Human Health and Disease. Semin. Immunol..

[77] Boyman O., Ramsey C., Kim D.M., Sprent J., Surh C.D. (2008). IL-7/Anti-IL-7 MAb Complexes Restore T Cell Development and Induce Homeostatic T Cell Expansion without Lymphopenia. J. Immunol..

[78] Inoue S., Unsinger J., Davis C.G., Muenzer J.T., Ferguson T.A., Chang K., Osborne D.F., Clark A.T., Coopersmith C.M., McDunn J.E. (2010). IL-15 Prevents Apoptosis, Reverses Innate and Adaptive Immune Dysfunction, and Improves Survival in Sepsis. J. Immunol..

[79] Hutchins N.A., Unsinger J., Hotchkiss R.S., Ayala A. (2014). The New Normal: Immunomodulatory Agents against Sepsis Immune Suppression. Trends Mol. Med..

[80] Zhao X., Qi H., Zhou J., Xu S., Gao Y. (2019). Treatment with Recombinant Interleukin-15 (IL-15) Increases the Number of T Cells and Natural Killer (NK) Cells and Levels of Interferon-γ (IFN-γ) in a Rat Model of Sepsis. Med. Sci. Monit..

[81] Bo L., Wang F., Zhu J., Li J., Deng X. (2011). Granulocyte-Colony Stimulating Factor (G-CSF) and Granulocyte-Macrophage Colony Stimulating Factor (GM-CSF) for Sepsis: A Meta-Analysis. Crit. Care.

[82] Mathias B., Szpila B.E., Moore F.A., Efron P.A., Moldawer L.L. (2015). A Review of GM-CSF Therapy in Sepsis. Medicine.

[83] Hall M.W., Knatz N.L., Vetterly C., Tomarello S., Wewers M.D., Volk H.D., Carcillo J.A. (2011). Immunoparalysis and Nosocomial Infection in Children with Multiple Organ Dysfunction Syndrome. Intensive Care Med..

[84] Meisel C., Schefold J.C., Pschowski R., Baumann T., Hetzger K., Gregor J., Weber-Carstens S., Hasper D., Keh D., Zuckermann H. (2009). Granulocyte-Macrophage Colony-Stimulating Factor to Reverse Sepsis-Associated Immunosuppression: A Double-Blind, Randomized, Placebo-Controlled Multicenter Trial. Am. J. Respir. Crit. Care Med..

[85] Quadrini K.J., Patti-Diaz L., Maghsoudlou J., Cuomo J., Hedrick M.N., McCloskey T.W. (2021). A Flow Cytometric Assay for HLA-DR Expression on Monocytes Validated as a Biomarker for Enrollment in Sepsis Clinical Trials. Cytom. Part B Clin. Cytom..

[86] Zhuang Y., Peng H., Chen Y., Zhou S., Chen Y. (2017). Dynamic Monitoring of Monocyte HLA-DR Expression for the Diagnosis, Prognosis, and Prediction of Sepsis. Front. Biosci. Landmark.

[87] Winkler M.S., Rissiek A., Priefler M., Schwedhelm E., Robbe L., Bauer A., Zahrte C., Zoellner C., Kluge S., Nierhaus A. (2017). Human Leucocyte Antigen (HLA-DR) Gene Expression Is Reduced in Sepsis and Correlates with Impaired TNFα Response: A Diagnostic Tool for Immunosuppression?. PLoS ONE.

[88] Zouiouich M., Gossez M., Venet F., Rimmelé T., Monneret G. (2017). Automated Bedside Flow Cytometer for MHLA-DR Expression Measurement: A Comparison Study with Reference Protocol. Intensive Care Med. Exp..

[89] Almansa R., Martín S., Martin-Fernandez M., Heredia-Rodríguez M., Gómez-Sánchez E., Aragón M., Andrés C., Calvo D., Rico-Feijoo J., Esteban-Velasco M.C. (2018). Combined Quantification of Procalcitonin and HLA-DR Improves Sepsis Detection in Surgical Patients. Sci. Rep..

[90] Liu F., Wang H.M., Wang T., Zhang Y.M., Zhu X. (2016). The Efficacy of Thymosin A1 as Immunomodulatory Treatment for Sepsis: A Systematic Review of Randomized Controlled Trials. BMC Infect. Dis..

[91] Romani L., Moretti S., Fallarino F., Bozza S., Ruggeri L., Casagrande A., Aversa F., Bistoni F., Velardi A., Garaci E. (2012). Jack of All Trades: Thymosin A1 and Its Pleiotropy. Ann. N. Y. Acad. Sci..

[92] Hotchkiss R.S., Monneret G., Payen D. (2013). Sepsis-Induced Immunosuppression: From Cellular Dysfunctions to Immunotherapy. Nat. Rev. Immunol..

[93] Dominari A., III D.H., Pandav K., Matos W., Biswas S., Reddy G., Thevuthasan S., Khan M.A., Mathew A., Makkar S.S. (2020). Thymosin Alpha 1: A Comprehensive Review of the Literature. World J. Virol..

[94] Wu J., Zhou L., Liu J., Ma G., Kou Q., He Z., Chen J., Ou-Yang B., Chen M., Li Y. (2013). The Efficacy of Thymosin Alpha 1 for Severe Sepsis (ETASS): A Multicenter, Single-Blind, Randomized and Controlled Trial. Crit. Care.

[95] Romani L., Bistoni F., Montagnoli C., Gaziano R., Bozza S., Bonifazi P., Zelante T., Moretti S., Rasi G., Garaci E. (2007). Thymosin A1: An Endogenous Regulator of Inflammation, Immunity, and Tolerance. Ann. N. Y. Acad. Sci..

[96] Feng Z., Shi Q., Fan Y., Wang Q., Yin W. (2016). Ulinastatin and/or Thymosin A1 for Severe Sepsis: A Systematic Review and Meta-Analysis. J. Trauma Acute Care Surg..

[97] Wang H., Liu B., Tang Y., Chang P., Yao L., Huang B., Lodato R.F., Liu Z. (2019). Improvement of Sepsis Prognosis by Ulinastatin: A Systematic Review and Meta-Analysis of Randomized Controlled Trials. Front. Pharmacol..

[98] Wysocka M., Montaner L.J., Karp C.L. (2005). Flt3 Ligand Treatment Reverses Endotoxin Tolerance-Related Immunoparalysis. J. Immunol..

[99] Toliver-Kinsky T.E., Lin C.Y., Herndon D.N., Sherwood E.R. (2003). Stimulation of Hematopoiesis by the Fms-like Tyrosine Kinase 3 Ligand Restores Bacterial Induction of Th1 Cytokines in Thermally Injured Mice. Infect. Immun..

[100] Patil N.K., Bohannon J.K., Luan L., Guo Y., Fensterheim B., Hernandez A., Wang J., Sherwood E.R. (2017). Flt3 Ligand Treatment Attenuates T Cell Dysfunction and Improves Survival in a Murine Model of Burn Wound Sepsis. Shock.

[101] Meng Y., Zhao Z., Zhu W., Yang T., Deng X., Bao R. (2017). CD155 Blockade Improves Survival in Experimental Sepsis by Reversing Dendritic Cell Dysfunction. Biochem. Biophys. Res. Commun..

[102] Chen R., Zhou L. (2021). PD-1 Signaling Pathway in Sepsis: Does It Have a Future?. Clin. Immunol..

[103] Zhang Y., Zhou Y., Lou J., Li J., Bo L., Zhu K., Wan X., Deng X., Cai Z. (2010). PD-L1 Blockade Improves Survival in Experimental Sepsis by Inhibiting Lymphocyte Apoptosis and Reversing Monocyte Dysfunction. Crit. Care.

[104] Patera A.C., Drewry A.M., Chang K., Beiter E.R., Osborne D., Hotchkiss R.S. (2016). Frontline Science: Defects in Immune Function in Patients with Sepsis Are Associated with PD-1 or PD-L1 Expression and Can Be Restored by Antibodies Targeting PD-1 or PD-L1. J. Leukoc. Biol..

[105] Shao R., Fang Y., Yu H., Zhao L., Jiang Z., Li C.S. (2016). Monocyte Programmed Death Ligand-1 Expression after 3-4 Days of Sepsis Is Associated with Risk Stratification and Mortality in Septic Patients: A Prospective Cohort Study. Crit. Care.

[106] Zhang Q., Qi Z., Liu B., Li C.S. (2018). Programmed Cell Death-1/Programmed Death-Ligand 1 Blockade Improves Survival of Animals with Sepsis: A Systematic Review and Meta-Analysis. Biomed Res. Int..

[107] Hotchkiss R.S., Colston E., Yende S., Crouser E.D., Martin G.S., Albertson T., Bartz R.R., Brakenridge S.C., Delano M.J., Park P.K. (2019). Immune Checkpoint Inhibition in Sepsis: A Phase 1b Randomized Study to Evaluate the Safety, Tolerability, Pharmacokinetics, and Pharmacodynamics of Nivolumab. Intensive Care Med..

[108] Chang K.C., Burnham C.A., Compton S.M., Rasche D.P., Mazuski R.J., SMcDonough J., Unsinger J., Korman A.J., Green J.M., Hotchkiss R.S. (2013). Blockade of the Negative Co-Stimulatory Molecules PD-1 and CTLA-4 Improves Survival in Primary and Secondary Fungal Sepsis. Crit. Care.

[109] European Medicines Agency (EMA) Opdivo (Nivolumab): An Overview of Opdivo and Why It Is Authorised in the EU. https://www.ema.europa.eu/en/documents/overview/mylotarg-epar-summary-public_en.pdf.

[110] Shindo Y., Unsinger J., Burnham C.-A., Green J.M., Hotchkiss R.S. (2015). Interleukin-7 and Anti–Programmed Cell Death 1 Antibody Have Differing Effects to Reverse Sepsis-Induced Immunosuppression. Shock.

[111] Pauken K.E., Sammons M.A., Odorizzi P.M., Manne S., Godec J., Khan O., Drake A.M., Chen Z., Sen D.R., Kurachi M. (2016). Epigenetic Stability of Exhausted T Cells Limits Durability of Reinvigoration by PD-1 Blockade. Science.

[112] Shindo Y., McDonough J.S., Chang K.C., Ramachandra M., Sasikumar P.G., Hotchkiss R.S. (2017). Anti-PD-L1 Peptide Improves Survival in Sepsis. J. Surg. Res..

[113] McBride M.A., Patil T.K., Bohannon J.K., Hernandez A., Sherwood E.R., Patil N.K. (2021). Immune Checkpoints: Novel Therapeutic Targets to Attenuate Sepsis-Induced Immunosuppression. Front. Immunol..

[114] Huang S.W., Chen H., Lu M.L., Wang J.L., Xie R.L., Zhao B., Chen Y., Xu Z.W., Fei J., Mao E.Q. (2018). Mycophenolate Mofetil Protects Septic Mice via the Dual Inhibition of Inflammatory Cytokines and PD-1. Inflammation.

[115] Zeng L., Kang R., Zhu S., Wang X., Cao L., Wang H., Billiar T.R., Jiang J., Tang D. (2017). ALK Is a Therapeutic Target for Lethal Sepsis. Sci. Transl. Med..

[116] Zhang Y.Y., Ning B. (2021). tao Signaling Pathways and Intervention Therapies in Sepsis. Signal Transduct. Target. Ther..

[117] Ge W., Hu Q., Fang X., Liu J., Xu J., Hu J., Liu X., Ling Q., Wang Y., Li H. (2019). LDK378 Improves Micro- and Macro-Circulation via Alleviating STING-Mediated Inflammatory Injury in a Sepsis Rat Model Induced by Cecal Ligation and Puncture. J. Inflamm..

[118] Vachharajani V.T., Liu T., Wang X., Hoth J.J., Yoza B.K., McCall C.E. (2016). Sirtuins Link Inflammation and Metabolism. J. Immunol. Res..

[119] Opal S.M., Ellis J.L., Suri V., Freudenberg J.M., Vlasuk G.P., Li Y., Chahin A.B., Palardy J.E., Parejo N., Yamamoto M. (2016). Pharmacological SIRT1 Activation Improves Mortality and Markedly Alters Transcriptional Profiles That Accompany Experimental Sepsis. Shock.

[120] Haak B.W., Prescott H.C., Wiersinga W.J. (2018). Therapeutic Potential of the Gut Microbiota in the Prevention and Treatment of Sepsis. Front. Immunol..

[121] Gaines S., Alverdy J.C. (2017). Fecal Micobiota Transplantation to Treat Sepsis of Unclear Etiology. Crit. Care Med..

[122] Keskey R., Cone J.T., DeFazio J.R., Alverdy J.C. (2020). The Use of Fecal Microbiota Transplant in Sepsis. Transl. Res..

[123] Li Q., Wang C., Tang C., He Q., Zhao X., Li N., Li J. (2015). Successful Treatment of Severe Sepsis and Diarrhea after Vagotomy Utilizing Fecal Microbiota Transplantation: A Case Report. Crit. Care.

[124] Khosrojerdi A., Soudi S., Hosseini A.Z., Eshghi F., Shafiee A., Hashemi S.M. (2021). Immunomodulatory and Therapeutic Effects of Mesenchymal Stem Cells on Organ Dysfunction in Sepsis. Shock.

[125] Laroye C., Gibot S., Huselstein C., Bensoussan D. (2020). Mesenchymal Stromal Cells for Sepsis and Septic Shock: Lessons for Treatment of COVID-19. Stem Cells Transl. Med..

[126] Keane C., Jerkic M., Laffey J.G. (2017). Stem Cell–Based Therapies for Sepsis. Anesthesiology.

[127] Laroye C., Gibot S., Reppel L., Bensoussan D. (2017). Concise Review: Mesenchymal Stromal/Stem Cells: A New Treatment for Sepsis and Septic Shock?. Stem Cells.

[128] Weiss A.R.R., Dahlke M.H. (2019). Immunomodulation by Mesenchymal Stem Cells (MSCs): Mechanisms of Action of Living, Apoptotic, and Dead MSCs. Front. Immunol..

[129] Messmer A.S., Que Y.A., Schankin C., Banz Y., Bacher U., Novak U., Pabst T. (2021). CAR T-Cell Therapy and Critical Care: A Survival Guide for Medical Emergency Teams. Wien. Klin. Wochenschr..

[130] Budde L.E., Zaia J.A. (2018). CD19 CAR-T Therapy and Sepsis: Dancing with the Devil. Blood.

[131] Zafer M.M., El-Mahallawy H.A., Ashour H.M. (2021). Severe COVID-19 and Sepsis: Immune Pathogenesis and Laboratory Markers. Microorganisms.

[132] van de Veerdonk F.L., Giamarellos-Bourboulis E., Pickkers P., Derde L., Leavis H., van Crevel R., Engel J.J., Wiersinga W.J., Vlaar A.P.J., Shankar-Hari M. (2022). A Guide to Immunotherapy for COVID-19. Nat. Med..

[133] Vincent J. (2021). COVID-19: It’s All about Sepsis. Future Microbiol..

[134] Noreen S., Maqbool I., Madni A. (2021). Dexamethasone: Therapeutic Potential, Risks, and Future Projection during COVID-19 Pandemic. Eur. J. Pharmacol..

[135] Kotsaki A., Pickkers P., Bauer M., Calandra T., Lupse M., Wiersinga W.J., Meylan S., Bloos F., Van Der Poll T., Slim M.A. (2022). ImmunoSep (Personalised Immunotherapy in Sepsis) International Controlled Randomised Clinical Trial: Study Protocol. BMJ Open.

[136] Prescott H.C., Kepreos K.M., Wiitala W.L., Iwashyna T.J. (2015). Temporal Changes in the Influence of Hospitals and Regional Healthcare Networks on Severe Sepsis Mortality. Crit. Care Med..

[137] König R., Kolte A., Ahlers O., Oswald M., Krauss V., Roell D., Sommerfeld O., Dimopoulos G., Tsangaris I., Antoniadou E. (2021). Use of IFNγ/IL10 Ratio for Stratification of Hydrocortisone Therapy in Patients With Septic Shock. Front. Immunol..

[138] Méndez Hernández R., Ramasco Rueda F. (2023). Biomarkers as Prognostic Predictors and Therapeutic Guide in Critically Ill Patients: Clinical Evidence. J. Pers. Med..

[139] Schuurman A.R., Sloot P.M.A., Wiersinga W.J., van der Poll T. (2023). Embracing Complexity in Sepsis. Crit. Care.

[140] Van der Poll T., Shankar-Hari M., Wiersinga W.J. (2021). The Immunology of Sepsis. Immunity.

[141] Shukla P., Rao G.M., Pandey G., Sharma S., Mittapelly N., Shegokar R., Mishra P.R. (2014). Therapeutic Interventions in Sepsis: Current and Anticipated Pharmacological Agents. Br. J. Pharmacol..

[142] Vincent J.-L., van der Poll T., Marshall J.C. (2022). The End of “One Size Fits All” Sepsis Therapies: Toward an Individualized Approach. Biomedicines.

[143] Liao Y.-E., Xu Y., Arnold K., Zhang F., Li J., Sellers R., Yin C., Pagadala V., Inman A.M., Linhardt R.J. (2023). Using Heparan Sulfate Octadecasaccharide (18-Mer) as a Multi-Target Agent to Protect against Sepsis. Proc. Natl. Acad. Sci. USA.

[144] Lin H.Y. (2020). The Severe COVID-19: A Sepsis Induced by Viral Infection? And Its Immunomodulatory Therapy. Chin. J. Traumatol..

[145] ImmunoSep Personalised Immunotherapy in Sepsis: A Precision Medicine Approach. https://immunosep.eu/.

